# Adjusting microbiome profiles for differences in microbial load by spike-in bacteria

**DOI:** 10.1186/s40168-016-0175-0

**Published:** 2016-06-21

**Authors:** Frank Stämmler, Joachim Gläsner, Andreas Hiergeist, Ernst Holler, Daniela Weber, Peter J. Oefner, André Gessner, Rainer Spang

**Affiliations:** Chair of Statistical Bioinformatics, University of Regensburg, Am BioPark 9, 93053 Regensburg, Germany; Institute of Clinical Microbiology and Hygiene, University Medical Centre, Franz-Josef-Strauß-Allee 11, 93053 Regensburg, Germany; Department of Haematology and Oncology, Internal Medicine III, University Medical Centre, Franz-Josef-Strauß-Allee 11, 93053 Regensburg, Germany; Chair and Institute of Functional Genomics, University of Regensburg, Am BioPark 9, 93053 Regensburg, Germany

**Keywords:** Microbial load, Spike-in bacteria, 16S rRNA gene sequencing, Standardization, Microbiome profiling, Bacterial communities, Community analysis

## Abstract

**Background:**

Next-generation 16S ribosomal RNA gene sequencing is widely used to determine the relative composition of the mammalian gut microbiomes. However, in the absence of a reference, this does not reveal alterations in absolute abundance of specific operational taxonomic units if microbial loads vary across specimens.

**Results:**

Here we suggest the spiking of exogenous bacteria into crude specimens to quantify ratios of absolute bacterial abundances. We use the 16S rDNA read counts of the spike-in bacteria to adjust the read counts of endogenous bacteria for changes in total microbial loads. Using a series of dilutions of pooled faecal samples from mice containing defined amounts of the spike-in bacteria *Salinibacter ruber*, *Rhizobium radiobacter* and *Alicyclobacillus acidiphilus*, we demonstrate that spike-in-based calibration to microbial loads allows accurate estimation of ratios of absolute endogenous bacteria abundances. Applied to stool specimens of patients undergoing allogeneic stem cell transplantation, we were able to determine changes in both relative and absolute abundances of various phyla, especially the genus *Enterococcus,* in response to antibiotic treatment and radio-chemotherapeutic conditioning.

**Conclusion:**

Exogenous spike-in bacteria in gut microbiome studies enable estimation of ratios of absolute OTU abundances, providing novel insights into the structure and the dynamics of intestinal microbiomes.

**Electronic supplementary material:**

The online version of this article (doi:10.1186/s40168-016-0175-0) contains supplementary material, which is available to authorized users.

## Background

The human intestinal tract is populated by an ecological community of microorganisms, the gut microbiome. This complex community plays an important role in health and disease [1–7] and varies greatly among individuals. Next generation sequencing of the 16S rRNA gene allows profiling of the bacterial and archaeal components of the gut microbiome at unprecedented precision and depth [8–10]. Computational tools such as QIIME [11] and mothur [12] cluster reads into operational taxonomic units (OTUs) [13], which may then be jointed into taxonomic groups at the genus, family, order, class, and phylum level.

Current studies focus on the relative abundance or proportions of OTUs [14, 15]. As an example, a specific OTU may contribute 5 % to microbiome A and 10 % to microbiome B corresponding to a ratio of 1:2. If we further assume that the total number of bacteria or microbial load of in A is four times larger than in B, the 5 % in A account for twice as many bacteria as the 10 % in B, thus bringing the actual ratio to 2:1.

Antibiotic treatment, diet, and/or disease affect both microbial loads and compositions. For example, Holler et al. [16] observed that the relative abundance of the genus *Enterococcus* in stool specimens collected from patients undergoing allogeneic stem cell transplantation (ASCT) can increase from undetectable levels prior to ASCT to up to 94 % after ASCT. More interestingly, this relative shift to *Enterococcus* was associated with an increased risk of acute gastrointestinal graft-versus-host disease (GI-GvHD). Without knowledge of total microbial load, however, it is impossible to infer whether this shift was the result of either an absolute increase in the number of *Enterococcus* or a decrease in the number of bacteria other than *Enterococcus*.

Application of synthetic spike-in standards allows for changing the profiles’ reference points. The reference point of relative abundances is a fixed aliquot of 16S rDNA. These profiles are insensitive to the microbial load of a stool specimen. Adding controlled amounts of spike-in material allows for rescaling the profiles such that the measured concentrations of the standard are constant across samples, making the spike-in standard the new reference point of the profiles and the profiles sensitive to microbial loads. Spike-in strategies featuring different GC contents and covering a wide concentration range in combination with appropriate normalization strategies have already been proposed to correct for library preparation and nuisance technical effects in the inference of gene expression levels from RNA-Seq experiments [17]. This approach, as well as similar schemes employed in proteomics [18] and metabolomics, [19] adds the spike-in standards to transcriptomes, proteomes and metabolomes only after cell lysis and extraction of mRNA, proteins and metabolites, respectively, and thus do not allow correction of variation originating from these critical experimental steps. Recently Jones et al. [20] suggested using whole cell spike-in controls for monitoring this technical variability in the field of microbiome research.

Extending their results, we here suggest the addition of exogenous viable spike-in bacteria to rescale the read counts of endogenous bacteria. We call this protocol *spike-in-based calibration to total microbial load* (SCML), and test it in a dilution experiment with defined absolute spike-in bacteria abundances against serially diluted background microbiomes. Moreover, we reconsider the emergence of *Enterococcus* as the predominant genus in ASCT using SCML.

## Results

### Choice of spike - in bacteria

We used *Salinibacter ruber* (*S. ruber*, GenBank ID: CP000159), an extreme halophilic bacterium found in hypersaline environments [21], *Rhizobium radiobacter* (*R. radiobacter*, GenBank ID: ASXY01000000), a non-phytopathogenic member of the Biovar I group of *Agrobacterium* found in the soil and the plant rhizosphere [22], as well as the thermo-acidophilic, endospore forming soil bacterium *Alicyclobacillus acidiphilus* (*A. acidiphilus*, GenBank ID: PRJDB697) [23]. These eubacteria belong to different phyla typically found in mammalian faecal microbiomes. They do not exist in the gut microbiome under physiological conditions and are well distinguishable from the bacteria in the gut using 16S rRNA gene sequencing. Whole bacteria are spiked-in at fixed amounts. 16S rRNA gene copy numbers per genome vary between these species (1, 4 and 6 rRNA gene copies per genome for *S. ruber*, *R. radiobacter* and *A. acidiphilus*, respectively). We quantify spike-in concentrations and measured concentrations relative to 16S rRNA gene copies. Hence, if we say that we spiked-in bacteria in the same amounts, this means that the number of 16S rRNA copies, but not necessarily the number of bacterial cells is identical.

### Design of spike-in calibration experiments

Experiments were performed with spike-in bacteria whose absolute abundance was defined by design in increasingly diluted gut microbiomes. The dilution simulates non-constant microbial loads.

*S. ruber*, *R. radiobacter* and *A. acidiphilus* were spiked into each of 36 aliquots of pooled murine stool samples. While *A. acidiphilus* and *R. radiobacter* were spiked into these samples at variable amounts, that of *S. ruber* was kept constant. *S. ruber* was used to measure microbial loads, while *A. acidiphilus* and *R. radiobacter* were used to validate the SCML approach. The precision of the spike-ins was independently validated using quantitative real time PCR (qRT-PCR). Importantly, this analysis also verified that all three bacteria were in fact not present in the pooled murine stool (Additional file 1: Table S1). Additional file 2: Table S2 summarizes the design of the validation experiment.

To validate the spike-in assay we compare calibrated ratios of observed reads with the expected ratios defined by the experimental design. The experimental design controls microbial loads at several levels:

(i) For each sample, we have expected total microbial loads defined by the stool dilution factor and the spike-in concentrations. (ii) For each of the two spike-ins *A. acidiphilus* and *R. radiobacter* we have expected within-species ratios of concentrations for every pair of samples (intra-OTU comparison). (iii) For every pair of samples we have expected inter-species ratios between the two spike-ins both within and across samples (inter-OTU comparison). (iv) For all taxonomic units of the background microbiome we have expected abundance ratios defined by the dilution factor and the spike-in concentrations.

### The three spike-in bacteria yield different read turnouts but correlate well with microbial loads

**Fig. 1:**
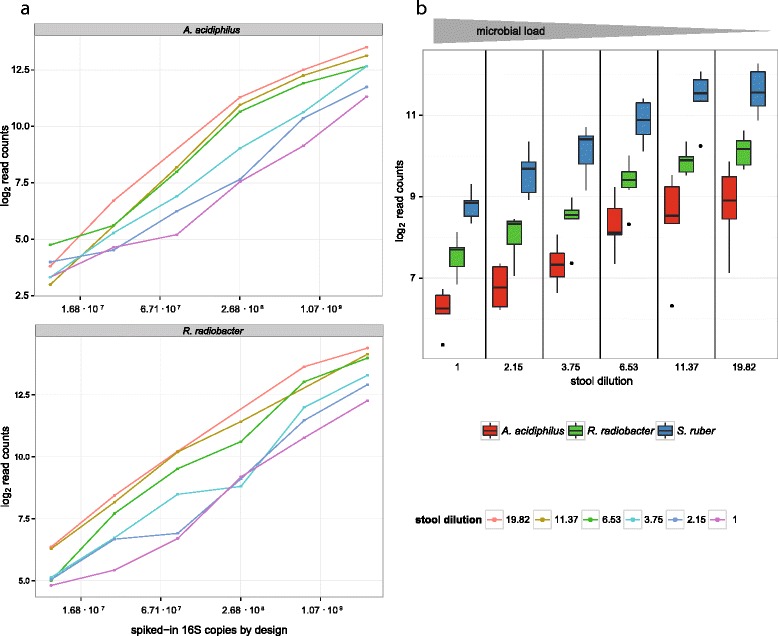
Log_2_ transformed read counts of the three spike-in bacteria as a function of total microbial load. *S. ruber* was added at a constant number of 16S rDNA copies, while *A. acidiphilus* and *R. radiobacter* were spiked in variably (cf. Additional file 2: Table S2). **a** Resulting read counts of *A. acidiphilus* and *R. radiobacter* versus spiked-in 16S rDNA copies at different background stool microbiota dilutions. Each dot represents a caecal specimen, while the colour specifies its dilution. **b** Boxplots showing the read counts of all three spike-in bacteria as a function of total microbial load. The log_2_ read counts of *S. ruber* are coloured blue, while *A. acidiphilus* and *R. radiobacter* are coloured red and green, respectively. Read counts of *A. acidiphilus* and *R. radiobacter* were adjusted by a factor corresponding to their difference of the predefined spike-in concentration to *S. ruber*. The x-axis is discrete and represents increasing stool dilution (bottom), as well as decreasing microbial load from left to right (grey arrowhead on the top)

Figure 1a shows linear relationships between the spiked-in 16S rDNA copies (x-axis in log_2_ scale) of *A. acidiphilus* and *R. radiobacter*, respectively, and the resulting log_2_ read counts. The total number of spike-in reads increases with dilution of the background microbiome. Simultaneously, as a constant amount of *S. ruber* was added to each sample, the portion of the spike-in bacteria increases (Fig. 1b). As a result, the read count assigned to a spike-in OTU is expected to inversely correlate with the total microbial load.

Figure 1b shows box plots of the log_2_ transformed read counts of *S. ruber, R. radiobacter* and *A. acidiphilus* as a function of microbial loads across all 36 samples. The counts were adjusted for their varying spike-in concentrations by design. For example, if in an experiment the concentration of the *A. acidiphilus* spike-in was only 50 % of that of *S. ruber*, the *A. acidiphilus* counts were doubled. After adjustment of *A. acidiphilus* and *R. radiobacter*, we observe an inverse correlation of log_2_ spike-in counts with the microbial load (reciprocal dilution factor) for all three spike-in bacteria (Fig. 1b). In detail there is a correlation of r = -0.834 for *S. ruber*, r = -0.795 for *R. radiobacter* (adjusted) and r = -0.725 for *A. acidiphilus* (adjusted). Additionally, we observe that the three bacteria have notably different read yields, with *S. ruber* showing the highest counts.

### SCML yields almost unbiased estimates of ratios of absolute abundances within taxonomic units

For comparing SCML to standard relative abundance analysis, we generated two data sets by scaling the read counts with respect to two different reference points: First, we scaled the observed read counts relative to the library sizes. This gives us the standard relative abundances (standard data). In a second data set we scaled the same counts relative to the spike-in reads of *S. ruber* (SCML data).

**Fig. 2:**
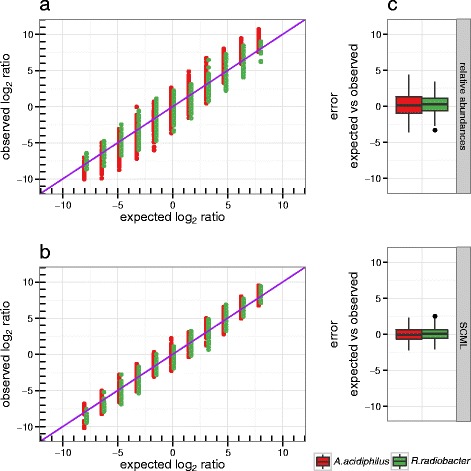
Comparison of log_2_ ratios derived from relative abundances and after applying SCML to *A. acidiphilus* and *R. radiobacter*. Observed log_2_ ratios versus expected log_2_ ratios of the spike-ins *A. acidiphilus* and *R. radiobacter* as derived from (**a**) relative abundances and (**b**) SCML by *S. ruber* for all pairwise sample comparisons. Both approaches were performed on the raw, not adjusted read counts of *A. acidiphilus* and *R. radiobacter*. The expected log_2_ ratios are calculated by the theoretical number of 16S rDNA copies predetermined in the design of the validation experiment (cf. Additional file 2: Table S2). The purple diagonal represents the identity, which represents the expected log_2_ ratios by design. The box plots in (**c**) show the error between the expected and observed log_2_ ratios for both approaches. The smaller this error, the better calibrated the ratios are

We first compared the data for *A. acidiphilus* and *R. radiobacter* separately. By design the expected ratio for *A. acidiphilus* and *R. radiobacter* between every pair of samples is known. Figure 2 shows the observed inter-sample ratios for both data sets as a function of expected ratios. Plot (a) was created using standard data, while plot (b) was created using SCML data. We observe a reduced systematic error in (b) when comparing the data trend to the identity line (purple). The standard data shows systematically overestimated ratios in both directions. SCML reduced this bias. Moreover, we observe a high variability of estimated ratios, which was almost cut in half by SCML (Fig. 2c).

**Fig. 3:**
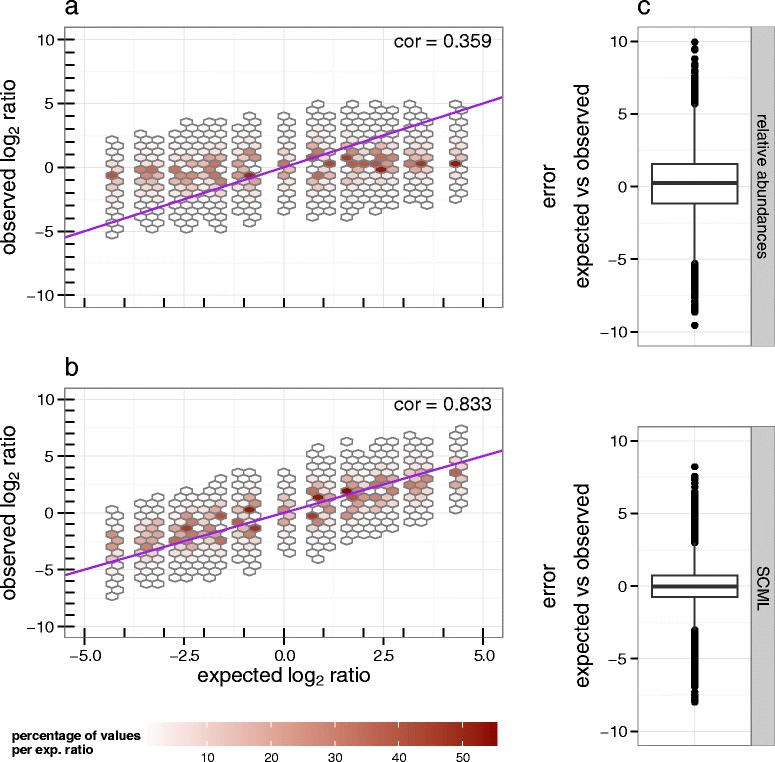
Comparison of log_2_ ratios derived from relative abundances and after applying SCML to all background OTUs. Observed log_2_ ratio versus expected log_2_ ratio of all background OTUs for all pairwise comparisons as derived from (**a**) relative abundances and (**b**) SCML by *S. ruber*. The data is binned to hexagons because of the high number of data points. The colour of each hexagon represents the percentage of counts at the corresponding level of expected log_2_ ratios contained in each bin. Bins that contributed to <0.05 % for each level of expected log_2_ ratio are omitted. The purple diagonal represents the identity, which represents the expected log_2_ ratios by design. The box-plots in (**c**) show the error between the expected and observed log_2_ ratios for both approaches. The smaller this error, the better calibrated the ratios are

We next analysed the ratios for the background OTUs. By design, experimentally controlled ratios can be calculated from the dilution factor of the background microbiome. In contrast to *A. acidiphilus* and *R. radiobacter* the ratios derived from relative abundances (standard data) of these OTUs is zero by experimental design. Figure 3 shows the distribution of observed background ratios as a function of corresponding expected ratios. Plot (a) was created using standard data, while plot (b) was created using SCML data. As expected, ratios of relative abundances cannot capture shifts in microbial loads that do not affect the composition (Fig. 3a). In line with the previous observations, we observe a reduction of estimation variance when using SCML (Fig. 3c). Correlations between expected and observed log_2_ ratios were 0.359 and 0.833 for the standard data and the SCML data, respectively.

### SCML allows more accurate estimation of ratios than calibrating for total 16S rRNA gene copies using qRT-PCR

**Fig. 4:**
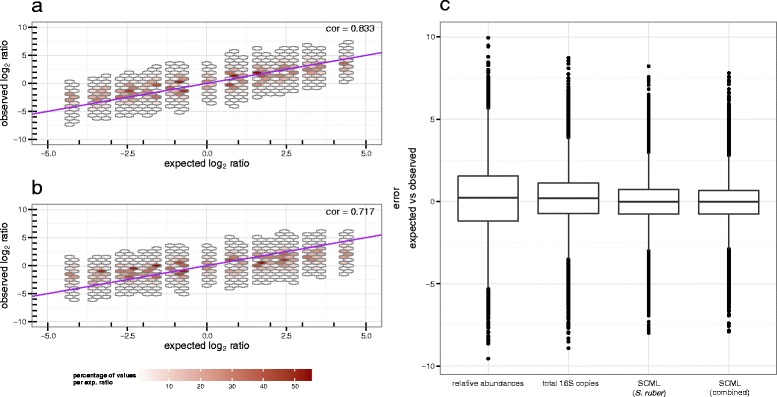
Comparison of SCML and normalization by qRT-PCR-derived total number of 16S rDNA copies to all background OTUs. Observed log_2_ ratio versus expected log_2_ ratio of all background bacteria OTUs for all pairwise sample comparisons after (**a**) SCML by *S. ruber* and (**b**) normalization by qRT-PCR derived total 16S rDNA copy number. The data is binned to hexagons because of the high number of data points. The colour of each hexagon represents the percentage of all counts at the corresponding level of expected log_2_ ratios contained in each bin. Bins that contributed to less than 0.05 percent for each level of expected log_2_ ratio are omitted. The purple diagonal represents the identity, which represents the expected log_2_ ratios by design. The box-plots in (**c**) summarize the error between the expected and observed log_2_ ratios for the four different approaches. The smaller this error, the better calibrated the ratios are. Variances of the log_2_ differences are 3.65, 2.01, 1.28 and 1.18 as derived from relative abundances, counts calibrated for differences in total number of 16S rRNA gene copies, SCML (by *S. ruber*) and combined SCML (by *S. ruber*, *A. acidiphilus* and *R. radiobacter*), respectively

Quantification of the total number of 16S rRNA gene copies by qRT-PCR may be used to determine microbial loads. To compare the practicability of the latter with SCML we used a SYBR Green-based qPCR assay to quantify 16S rDNA (Additional file 1: Table S1). Figure 4 shows observed and expected log_2_ ratios for background OTUs using either (a) SCML or (b) rescaling to constant total 16S rRNA gene copies. It is apparent that observed ratios derived from SCML show higher concordance with the expected ratios regarding estimation bias and variance. Correlations between expected and observed log_2_ ratios were 0.717 and 0.833 for the qPCR and the SCML approach, respectively. These findings are also supported by an overall lower error between the observed and expected log_2_ ratios when derived from SCML (Fig. 4c). However, it has to be acknowledged that the SYBR Green-based quantification method of the bacterial load has not been explicitly compared to probe-based formats, so any limitations/imprecisions possibly resulting from the use of this universal detection format were not taken into account.

### Combining multiple spike-in bacteria reduces estimation errors

Figure 1b shows that the adjusted counts of all three spike-in bacteria reciprocally correlated with microbial loads. We next investigated whether taking the sum of all three spike-in read counts further improves the estimates. Since *A. acidiphilus* and *R. radiobacter* were spiked in variable amounts we had to adjust their counts prior to using them for calibration. For example, if in an experiment the concentration of the *A. acidiphilus* spike-in was only 50 % of that of *S. ruber*, the *A. acidiphilus* counts were doubled. We then used the sum of adjusted counts of all three spike-ins for calibration and repeated the analysis of the previous section. Figure 4c shows box-plots of the error between expected and observed log_2_ ratios for background OTUs based on relative abundances, read counts normalized by total 16S rDNA copies, as well as based on the SCML data with *S. ruber* only and the combined counts of all three spike-ins, respectively. The smaller this error, the better calibrated are the ratios of absolute abundances. Variances of these errors are 3.65, 2.01, 1.28 and 1.18, respectively. Thus, combined spike-ins yield a slightly increased precision compared to single spike-in usage. Correlations between expected and observed log_2_ ratios were 0.833 and 0.845 for the SCML and the combined SCML approach, respectively.

### Calibration to microbial loads reveals absolute increase of *Enterococcus* in the intestine during allogeneic stem cell transplantation

Finally, we show that SCML expands our understanding of human microbiomes and their role in disease. Recently, a marked early loss of gastrointestinal microbiome diversity and an increase in relative abundance of members of the genus *Enterococcus* have been observed in the course of allogeneic stem cell transplantation (ASCT) and found to increase the risk of developing acute GI-GvHD [16, 24, 25]. Since the data had been generated without spike-in bacteria, it had not been possible to conclude whether the observed increase in relative abundance of *Enterococcus* was the result of an increase in absolute abundance of *Enterococcus* or of a decrease in abundance of other bacterial species.

**Fig. 5:**
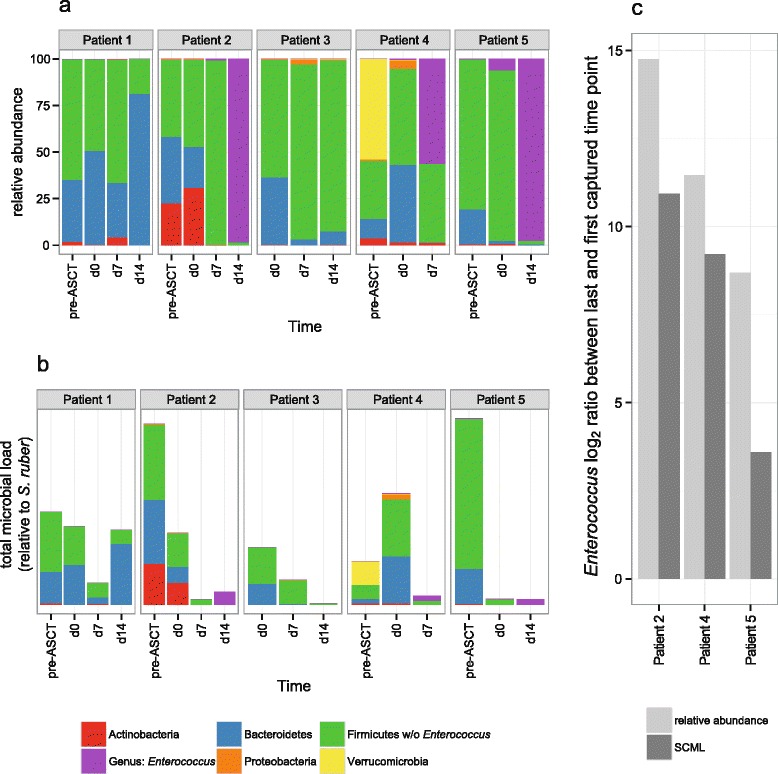
Bacterial abundances in stool specimens of ASCT patients. Specimens were collected prior to administration of prophylactic antibiotics and radio-chemotherapeutic conditioning (pre-ASCT) and on days 0, 7 and 14 after ASCT (d0, d7, d14). **a** Microbial composition given as in relative abundances; (**b**) read counts scaled to a uniform count of the spike-in *S. ruber* and (**c**) log_2_ ratios of *Enterococcus* of the last time point to pre-ASCT of patients 2, 4 and 5 as derived from relative abundances (light grey) and SCML (dark grey). In (**a**) and (**b**) the reads of the three spike-in bacteria are omitted. Additionally, the reads that contributed to the genus of *Enterococcus* are excluded from the Firmicutes phylum and coloured separately (purple)

Here we report on five patients, whose stool microbiomes were monitored prior to ASCT or on days 0 (d0), 7 (d7), and 14 (d14) after ASCT, respectively, using the proposed spike-in approach. Figure 5a shows the familiar diagram of relative microbiome composition without taking the spike-in bacteria into consideration. Reads contributing to the genus of *Enterococcus* are reported at genus resolution, while all other bacteria are shown on phyla resolution. In line with Holler et al. [16], we observe dramatic relative increases in *Enterococcus* abundance on days 7 and 14 after ASCT in three of the five patients. By scaling read counts to an even microbial load using the *S. ruber* counts, we observe marked changes in the microbial loads in the course of the treatment (Fig. 5b). Patient 5, for instance, shows an almost tenfold reduction of microbial load on day 14 after ASCT (*S. ruber* reads 4721) compared to pre-ASCT (*S. ruber* reads 515). In our study, specimens dominated by *Enterococcus* generally have low microbial loads (Fig. 5b). We also observe an absolute increase in abundance of the genus *Enterococcus* in these microbiomes relative to the specimens collected before ASCT (Fig. 5c). Patients 2, 4 and 5 showed log_2_ ratios of *Enterococcus* between the last and first time point of 10.93, 9.22 and 3.60, respectively, employing SCML, compared to log_2_ ratios of 14.76, 11.46 and 8.69 based on standard data. This suggests that *Enterococcus* dominance is in fact associated with both a decrease in microbial load and a rise in absolute abundance of *Enterococcus*.

## Discussion

Here we suggest the use of spike-in bacteria to calibrate multiple intestinal microbiome profiles to microbial loads (SCML). We employed *A. acidiphilus*, *R. radiobacter* and *S. ruber* as spike-in bacteria and demonstrated their excellent suitability for a comprehensive and informative profiling of gut microbiomes. Usually, these three bacteria are absent in the intestinal microbiomes of mammals, and their unique 16S rRNA gene sequences cannot be mistaken for those of bacteria found in the gastrointestinal tract. All three bacteria are valid reporters of the actual microbial load. Thus SCML adds a new perspective to gut microbiome profiling that expands the common relative microbiome composition analysis.

Variability in microbial loads of intestines is a genuine and potentially clinically relevant biological feature that remains underutilised in standard protocols. On a more technical side, adding whole cells prior to lysis enables control for DNA recovery and pyrosequencing errors as a side benefit. Following this, the addition of exogenous spike-ins could also enhance other studies like whole-genome sequencing, qPCR-based quantification of pathogens as well as approaches using alternative marker genes [20, 26, 27].

Bacterial species compete for nutrients and can mutually displace each other, while others can only live in symbiosis. These dynamics of the intestinal ecosystem shape the structure of microbiome profiles [28, 29]. Mutually displacing, e.g. concurrent or antagonistic, species display anti-correlated profiles, while those of symbiotic species are correlated [30]. This theoretical consideration holds true for absolute numbers of bacteria. Interpreting the correlation structure on the basis of relative numbers can be misleading. If one species grows in absolute number, this will lead (i) to an increase of its fraction within the microbiome and (ii) to a decrease of the fractions of all other species. Hence, every change of a single species affects relative counts for all other species generating notorious anti-correlation between profiles of different species due to compositionality [31, 32]. Importantly, this effect is independent of ecological processes like displacement and symbiosis. Thus, profiles calibrated to ratios of total microbial loads provide a less disturbed assessment of the dynamics of the intestinal ecosystem.

We observed different sequence read yields for the three spike-in bacteria even upon addition of identical numbers of 16S rDNA gene copies to mouse faeces sharing the same microbial load. Fortunately, this problem should only arise with comparisons of different species. As demonstrated, it does not affect the estimation of intra-species ratios between samples, where species-specific yields cancel.

There is a difference between absolute quantification and calibration of ratios of absolute abundances. The former needs calibration to a defined unit such as bacteria per volume. External spike-ins do not enable absolute quantification due to e.g. variable lysis efficiency across intestinal bacteria and variable 16S rDNA copy numbers. In ratios the unit cancels. Hence, if in a comparison of the same OTU in two samples SCML calibrated values show a ratio of 2, then the OTU is in fact represented (almost) twice as often. With standard relative data this is not the case, when microbial loads in these samples differ. Importantly, ratios between different OTUs are not calibrated by SCML. We can thus calibrate microbiome profiles to ratios of absolute abundance but not to absolute quantities of bacteria themselves.

A drawback of the spike-in approach is the propagation of PCR amplification errors from the spike-in bacterium to all other taxonomic units. Indeed, the spike-in counts can be affected by PCR amplification or sequencing errors. The earlier these errors occur, the more they could influence the final read tallies. By using these reads to calibrate microbial ratios, this error-derived variance propagates to all other taxonomic units. The calibration reduces bias, but inflates variance. One may attenuate this undesired effect by using multiple spike-in bacteria of fixed concentrations across samples and averaging or summing their counts as shown here.

## Conclusion

In summary, we suggest that scaling of read counts to total microbial load and thereby calibrating the ratios of taxonomic units become standard routine in microbiome analysis, complementing the classical analysis of relative microbiome composition. SCML allows for accurate inter-sample comparison of microbiome profiles even in the presence of variation in microbial loads. Further, compared to the measurement of total number 16S rDNA gene copies by qPCR, the spike-in bacteria are able to control additionally to DNA isolation and PCR amplification the sequencing process. Finally, our method allows a more robust identification of differentially abundant OTUs by enabling more accurate estimations of OTU-specific ratios.

## Methods

### Spike-in bacteria

In this study we used *Salinibacter ruber DSM 13855*^*T*^, an extreme halophilic bacterium found in hypersaline environments [21], *Rhizobium radiobacter DSM 30147*^*T*^, a non-phytopathogenic member of the Biovar I group of *Agrobacterium* found in soil and the plant rhizosphere [22], as well as the thermo-acidophilic, endospore forming soil bacterium *Alicyclobacillus acidiphilus DSM 14558*^*T*^ [23]. All bacteria were purchased from the DSMZ (German Collection of Microorganisms and Cell Cultures GmbH, Braunschweig, Germany). These eubacteria belong to different phyla typically found in mammalian faecal microbiomes, contributing to Bacteroidetes/Chlorobi group, Proteobacteria, and Firmicutes, respectively. They do not exist in the gut microbiome under physiological conditions and are well distinguishable from bacteria commonly found in the gut using 16S rRNA gene sequencing. *S. ruber* and *R. radiobacter* are gram-negative bacteria, whereas *A. acidiphilus* is a spore-forming gram-positive bacterium. The difference in the chemical constitution of the cell wall accounts for a specific susceptibility to the cell lysis protocol used. Spike-in bacteria were harvested in the late logarithmic/early stationary growth phase by centrifugation and subsequently resuspended in 5 ml of sterile PBS buffer. Bacterial densities in suspensions were quantified by OD600 measurement using empirical conversion factors determined by direct microscopic cell counting. Accordingly, 1 OD600 unit corresponds to 4.6 x 10^9^ cells/ml for *S. ruber*, 1.4 x 10^9^ cells/ml for *R. radiobacter*, and 1.2 x 10^9^ cells/ml for *A. acidiphilus*, respectively. 16S rRNA gene copy numbers per genome for the spike-in bacteria were obtained from the rrnDB database [33]. Six pools of bacterial mock communities containing *S. ruber*, *R. radiobacter* and *A. acidiphilus* were generated according to the scheme provided in Additional file 3: Table S3.

### Sample preparation and DNA extraction

#### Mouse specimens

For the validation experiment, cecum contents were collected from three 12-week-old male C57BL/6J mice (200 mg wet weight each), immediately suspended into 1 ml of PBS, homogenized by means of the TissueLyser II (QIAGEN, Hilden, Germany), pooled, adjusted with PBS to a total volume of 4 ml, and split into seven aliquots of 550 μl each. Six of these aliquots were diluted five times according to the scheme provided in Additional file 2: Table S2. Aliquot 7 was used as a non-spike control.

**Fig. 6:**
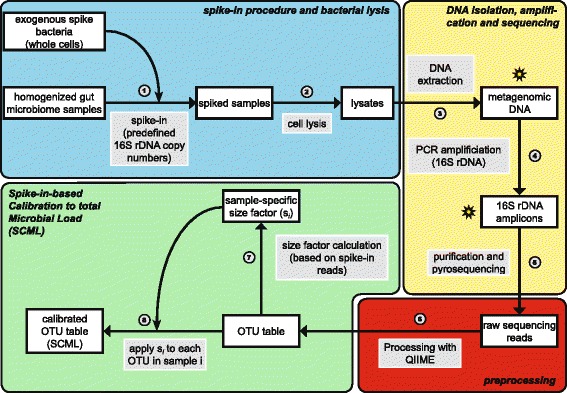
Procedural overview of proposed spike-in procedure and the spike-in-based calibration to total microbial load (SCML). The overview is divided into four sections: spike-in procedure and bacterial lysis (blue), DNA isolation, amplification and sequencing (yellow), pre-processing (red) and the actual spike-in-based calibration to microbial load (green). White-filled boxes depict procedural intermediates, while grey-filled boxes depict the different procedural steps. Each step is numbered. In the first step (1) whole cells of exogenous spike bacteria corresponding to a fixed number of 16S rDNA copies are added to homogenized microbiome samples. Bacterial lysis is performed on the resulting spiked samples (2). Metagenomic DNA is extracted from the lysates (3) and PCR amplified using 16S rDNA specific primers (4), creating 16S rDNA amplicons. These amplicons are purified and pyrosequencing is performed (5). The resulting raw read counts are pre-processed with QIIME (quality filtering, demultiplexing and closed reference OTU picking) to generate OTU read count tables (6). Based on the read counts associated with single or multiple reference spike-in bacteria, a size factor s_i_ for each sample i is calculated and applied to each OTU of this particular sample i (8, see methods section). This leads to an OTU read count table calibrated to differences in microbial load. These read counts can be utilized to more accurately assess changes between different samples. All depicted steps are described in detail in the methods section. Stars indicate points in the procedure at which qPCR is performed to identify possible errors in DNA isolation (metagenomic DNA) or PCR amplification (16S rDNA amplicons).

Sixty microliters of the corresponding spike-bacteria pool (whole cells) containing the desired number of 16S rDNA copies (see Additional file 3: Table S3) were added to 250 μl of all prepared, unlysed stool dilutions (see Fig. 6, step 1) according to the scheme provided in Additional file 2: Table S2. Then, 180 μl of Bacterial Lysis Buffer (Roche, Mannheim, Germany) and 20 μl Proteinase K (Fermentas GmbH, Sankt Leon-Rot, Germany) were added. Samples were incubated at 65 °C for 10 min followed by five cycles of freezing in liquid nitrogen (1 min) and boiling in hot water (95 °C, 1 min). Following the addition of 400 μl of Bacterial Lysis Buffer and a mixture of 0.1-mm and 2.5-mm beads, samples were treated for 2 min at 30 Hz in the TissueLyser II. Subsequently, samples were heated at 95 °C for 15 min and centrifuged at 4 °C to pellet stool particles and beads (see Fig. 6, step 2). The final volume was adjusted to 1 ml and DNA was extracted (see Fig. 6, step 3) by means of the MagNA Pure 96 instrument employing the MagNA Pure 96 DNA and Viral NA Large Volume Kit (Roche). Nucleic acids were quantified using the NanoDrop ND-1000 (Thermo Scientific, Wilmington, DE, USA).

#### Human ASCT specimens

With approval of the Ethics Committee of the University Medical Centre of Regensburg and after receipt of signed informed consent forms, stool specimens were collected at four different time points: prior to administration of prophylactic antibiotics and radio-chemotherapeutic conditioning, on days 0, 7, and 14, respectively, after ASCT. Stool specimens were stored at -80 °C until analysis. Fifty mg (wet weight) of each stool specimen were suspended into 250 μl PBS and subsequently subjected to DNA extraction as described above. Spiking of *A. acidiphilus*, *S. ruber* and *R. radiobacter*, and 454-pyrosequencing were performed according to the validation protocol described above. For these experiments, bacterial cells of *S. ruber*, *R. radiobacter* and *A. acidiphilus* equal to 3.0 x10^8^, 5.0x10^8^, 1.0x10^8^ 16S rDNA copies, respectively, were spiked into each crude sample.

### Amplification of V3-V6 16S rDNA variable region and 454 pyrosequencing

Spike bacteria-specific qPCR was performed for all specimens (mice and human) to identify errors in DNA isolation before undergoing amplification and pyrosequencing (see Fig. 6).

A total of 25 ng metagenomic DNA was used as a template to amplify the V3-V6 variable regions of the 16S rRNA gene. PCR was performed using primer pair 341 F-1061R containing Lib-L adaptors and Roche standard mulitiplex identifiers (MIDs) in a final volume of 40 μl containing 0.088 μM of each primer, 2 mM MgCl_2_, and 1 U Platinum Taq DNA Polymerase (Life Technologies). The PCR amplification (see Fig. 6, step 4) was carried out over 30 cycles (30s at 95 °C, 45 s at 64 °C, 45 s at 72 °C) with an initial 5-min hot start at 95 °C and a final extension step (7 min at 72 °C). The resulting 790-bp amplicons were analysed by standard agarose gel electrophoresis on a 1.5 % (w/v) gel. The amplicons were extracted from agarose gels using the QIAquick Gel Extraction Kit (Qiagen, Hilden, Germany) and purified with Agencourt AMPure XP beads (Beckman Coulter, Krefeld, Germany). Copy numbers of amplicons containing LibL-adaptors were determined using the KAPA Library Quant 454 Titanium/Lib-L Universal Kit (KAPA Biosystems, Wilmington, DE, USA) and pooled to a normalized library with a concentration of 1 x 10^7^ adaptor-labeled amplicon molecules/μl for each sample. This library was subjected to sequencing (see Fig. 6, step 5) using the GS FLX+ system (454/Roche) and the GS FLX Titanium LV emPCR Kit (Lib-L) applying 0.4 copies per bead. Sequencing was performed on a full PTP according to manufacturer’s protocol using the GS-FLX Titanium Sequencing Kit XL+ and the acyclic flow pattern B. Sequencing raw data was processed with gsRunProcessor v2.9 (Roche) using quality filtering as defined by the default LongAmplicons 3 pipeline resulting in 895 Mb from 1,313,653 passed filter wells with a median read length of 706 bases.

### Quantification of 16S rRNA gene copy number by qRT-PCR

#### Primer design and validation

Primers and probes for quantification of eubacterial 16S rDNA copies (Additional file 4: Table S4) were designed and evaluated *in silico* based on the RefNR sequence collection of the SILVA reference database release 119 [34] containing 534,968 16S rRNA sequences. The overall SILVA database coverage of universal 16S rDNA quantification primers 764 F and 907R allowing one primer mismatch was 86 %. Allowing no primer mismatches, specificity of primers and probes targeting *S. ruber, R. radiobacter* and *A. acidiphilus* DNA exhibited specificities of 100 %. Specificity of primers and probes were further evaluated in silico using the blastn algorithm against the nucleotide collection (nt) database. Concentration of primers were optimized by titration in the range of the kit manufacturer’s recommendations after PCR amplification of 16S rDNA targets from DNA extracts of human and murine faecal specimens. Samples were spiked prior to DNA extraction with defined cell counts of *S. ruber, R. radiobacter* and *A. acidiphilus,* which were quantified microscopically using a modified Neubauer counting chamber. PCR products were screened for nonspecific bands by agarose gel electrophoresis (probe based assays) or agarose gel and melting curve analysis (SYBR Green I based assays). Specificity was further evaluated by quantitative real-time PCR amplification of total 16S rDNA and 16S rDNA of spike-in bacteria from ten non-spiked murine and human DNA extracts.

#### Quantification of total 16S rDNA

To verify the experimental design, 16S rRNA gene copies of total and spike-in bacteria were determined by qRT-PCR on a LightCycler 480 II Instrument (Roche). Primers and probes used are shown in Additional file 4: Table S4. PCR reactions included 1 μM each of eubacterial 16S rRNA gene primers 764 F and 907R (quantification primers) and the LightCycler 480 SYBR Green I Master Kit (Roche). Quantification standards were generated by cloning complex PCR amplicon mixtures that were generated from a caecal microbiome DNA preparation of wild type C57BL/6J mice (using primers 341 F and 1061R) into the pGEM-T.Easy vector (Promega, Madison, WI, USA). Cloning of PCR amplicon mixtures was carried out to mimic a complex murine microbiota with respect to qPCR amplification efficiency in analyzed samples as far as possible. Quantification PCR was conducted over 40 cycles (95 °C for 10s, 60 °C for 15 s and 72 °C for 15 s) with an initial 10-min hot start at 95 °C.

#### Quantification of 16S rDNA of spike-in bacteria

16S rRNA gene copy numbers of the spike-in bacteria *S. ruber, R. radiobacter* and *A. acidiphilus* were determined with 16S rDNA-targeted species-specific primers and hydrolysis probes (see Additional file 4: Table S4). Quantification PCR was conducted using the LightCycler 480 Probes Master kit (Roche) in a 20-μl reaction volume containing 4 mM MgCl_2_, 0.25 μM of each primer, and 0.1 μM probes. Quantification standards were constructed by cloning full length 16S PCR amplicons of all spike-in bacteria (amplified using 27 F and 1492R primers) into pGEM-T.Easy. Quantification PCR was conducted over 40 cycles (95 °C for 30s, 60 °C for 30 s and 72 °C for 30s) with an initial 10-min hot start at 95 °C.

### Computational analysis

We used a combination of QIIME [11] (v1.8.0) and R version 3.2.0 [35] with installed Bioconductor package [36] to process the read data. Reads were filtered for quality using QIIME’s *split_libraries.py* script (see Fig. 6, step 6) with default parameters except minimum and maximum read length, which were set to 400 bp and 800 bp, respectively. This read length threshold covered 99.99 % of all sequencing reads. The filtered reads were mapped to OTUs built on the SILVA [34] database (release 111) using QIIME’s *pick_closed_reference_otus.py* script (see Fig. 6, step 6) with default parameters. The reference database OTUs used here constituted computationally built clusters of the SILVA SSU (small subunit) ribosomal RNA database. The clustering (see Fig. 6, step 6) was achieved by UCLUST 1.2.20 [37] and provided by the QIIME team (available at http://qiime.org/home_static/dataFiles.html). Since reads from the three spike-in bacteria mapped to multiple OTUs, due to multiple reference OTUs encoding for the same spike-in genus, we deleted all but one OTU encoding for each spike-in from the database before mapping, to accumulate all reads from the spike-in to just this one OTU. The used reference sequences for these three OTUs are available in Additional file 5. Raw sequencing data of the dilution experiment is deposited in the European Nucleotide Archive (ENA) under the study accession number PRJEB11953, at http://www.ebi.ac.uk/ena/data/view/PRJEB11953. Details of the sample design are shown in Additional file 2: Table S2. Relative abundances were calculated by dividing each OTU read count by total read count of the corresponding sample.

Ratios of absolute abundances were calculated by using the expectation that the counts of reference spike-ins are inversely correlated to total microbial load of the samples under investigation. Let $\overline{s}$ be the mean read count of the reference spike-in *S. ruber* over all samples (see Fig. 6, step 7). The read count of every OTU in a sample_i_ is rescaled by a factor s_i_ that is calibrated such that the spike-in count is equal to $\overline{s}$ in every sample (see Fig. 6, step 8). SCML can be performed by the use of an individual spike-in bacterium or the sum of all reads obtained for multiple spike-in bacteria. For further analysis, the counts are log_2_ transformed.

To compare ratios derived from relative abundances and those derived by SCML, we calculate log_2_ ratios between every pair of samples for each method as a symmetrical measure of difference. Ratios of relative abundances are calculated by dividing the relative abundances of each OTU by its relative abundance in the compared sample, whereas ratios for SCML are calculated by means of the spike-in calibrated read counts (SCML data). If for example OTU A shows relative abundances of 20 % and 40 % in samples 1 and 2, respectively, the corresponding ratio for this comparison would be 0.4/0.2 = 2, i.e. the abundance of OTU A in sample 2 is two times higher than in sample 1. The corresponding log_2_ ratio would be log_2_ (2) = 1. Both ratios are calculated separately for each OTU.

For the combination approach of SCML, the read counts of *A. acidiphilus* and *R. radiobacter* were adjusted by their difference in the predefined spike-in concentration (Additional file 2: Table S2) towards *S. ruber*. If for example *A. acidiphilus* was added by design in half the concentration compared to *S. ruber*, then all reads by *A. acidiphilus* were multiplied by two. The adjusted read counts of *A. acidiphilus*, *R. radiobacter* and the raw read counts of *S. ruber* were summed up to one artificial entity. These summed reads were used in the same fashion as the *S. ruber* read counts in the single spike-in calculation. For the dilution experiment the adjustment of *A. acidiphilus* and *R. radiobacter* read counts was necessary, because both spike-ins were added in varying amounts in this experiment. In an application of our spike-in procedure (e.g. ASCT specimens in this study) all spike-in bacteria cells are added at fixed amounts. Therefore, an adjustment of the spike-in read counts before the combination would be obsolete.

## Abbreviations

*A. acidiphilus*, *Alicyclobacillus acidiphilus;* ASCT, allogeneic stem cell transplantation; GI-GvHD, gastrointestinal graft-versus-host disease; OTU, operational taxonomic unit; qRT-PCR, quantitative real-time polymerase chain reaction; *R. radiobacter*, *Rhizobium radiobacter; S. ruber*, *Salinibacter ruber;* SCML, spike-in-based calibration to microbial load

## Additional files

Additional file 1: Table S1: Species-specific and total 16S rDNA quantitative realtime-PCR measurements. (XLSX 13 kb) Additional file 2: Table S2: Design of dilution experiment. (XLSX 16 kb) Additional file 3: Table S3: Pools of bacterial mock communities containing *S. ruber*, *A. acidiphilus* and *R. radiobacter*. (XLSX 13 kb) Additional file 4: Table S4: Primers and hydrolysis probes used in this study. (XLSX 13 kb) Additional file 5: 16S rDNA reference sequences of *S. ruber*, *A. acidiphilus* and* R. radiobacter*. (FASTA 4 kb) Additional file 6: R-Script for reproduction of analysis and figures of this study. (R 39 kb) Additional file 7: Dilution experiment mapping file as used for reproduction. (TXT 3 kb) Additional file 8: ASCT experiment mapping file as used for reproduction. (TXT 1 kb) Additional file 9: Dilution experiment OTU table as used for reproduction. (TXT 460 kb) Additional file 10: ASCT experiment OTU table as used for reproduction. (TXT 621 kb) Additional file 11: Total 16S rDNA qRT-PCR measurements as used for reproduction. (CSV 1 kb) Additional file 12: Spike-in concentrations by design as used for reproduction. (CSV 1 kb)
